# Evaluation of CHA_2_DS_2_-VA Score and Systemic Inflammatory Indexes in Patients with Nonvalvular Atrial Fibrillation: A Case–Control Study

**DOI:** 10.3390/jcm14134601

**Published:** 2025-06-29

**Authors:** Abdulkadir Cakmak, Sirin Cetin, Ercan Kahraman, Meryem Cetin

**Affiliations:** 1 Department of Cardiology, Faculty of Medicine, Amasya University, Amasya 05200, Türkiye; 2 Department of Biostatistics, Faculty of Medicine, Amasya University, Amasya 05200, Türkiye; cetinsirin55@gmail.com; 3 Department of Cardiovascular Surgery, Faculty of Medicine, Amasya University, Amasya 05200, Türkiye; dr.ercankahraman@gmail.com; 4 Department of Microbiology, Faculty of Medicine, Amasya University, Amasya 05200, Türkiye; meryemcetin55@yahoo.com

**Keywords:** nonvalvular atrial fibrillation, novel CHA_2_DS_2_-VA score, novel inflammatory indexes

## Abstract

**Background/Objectives:** Nonvalvular atrial fibrillation (NVAF) is a prevalent arrhythmia associated with elevated risks of stroke, systemic embolism, and mortality. Emerging evidence underscores the pivotal role of inflammation in NVAF pathogenesis. The CHA_2_DS_2_-VA score is currently the most powerful tool used in the management of patients with atrial fibrillation, and integrating novel inflammatory biomarkers—neutrophil-to-lymphocyte ratio (NLR), platelet-to-lymphocyte ratio (PLR), systemic immune-inflammation index (SII), and systemic inflammation response index (SIRI)—into this score may enhance prognostic accuracy and guide personalized therapy. **Methods:** In this observational case–control study, a cohort of 330 NVAF patients and 201 controls, inflammatory and biochemical parameters were measured and compared, we employed multivariate logistic regression and ROC analyses to validate the discriminative power of novel inflammatory indexes and novel CHA_2_DS_2_-VA score, setting a new benchmark for biomarker integration in NVAF management. **Results:** Inflammatory indexes (NLR, PLR, SII, SIRI) were significantly higher in NVAF patients compared to controls (*p* < 0.001). Multivariate analysis identified NLR (OR = 4.02), PLR (OR = 1.04), SII (OR = 1.01), and SIRI (OR = 1.87) as independent NVAF risk markers. The CHA_2_DS_2_-VA score showed the strongest association with NVAF (OR = 5.55), and an optimal cutoff of ≥2 yielded 88.18% sensitivity and 74.63% specificity. **Conclusions:** Inflammatory markers NLR, PLR, SII, and SIRI, when assessed alongside the CHA_2_DS_2_-VA score, offer significant and complementary prognostic insight for patients with NVAF. These findings support the integration of inflammatory indexes into routine clinical risk assessment models to enhance early identification of high-risk individuals and inform personalized therapeutic strategies. Moreover, our findings provide a rationale for developing composite risk scores in future studies that integrate inflammatory biomarkers with the CHA_2_DS_2_-VA score (e.g., a CHA_2_DS_2_-VA-Inflammation Score). Further large-scale, longitudinal studies are warranted to validate these results and explore the benefits of inflammation-targeted interventions.

## 1. Introduction

Nonvalvular atrial fibrillation (NVAF) is a common arrhythmia involving disorganized atrial activity, resulting in heightened stroke risk and mortality [1]. Its rising prevalence parallels aging populations and comorbidities like hypertension and diabetes [2,3]. Inflammatory processes, including oxidative stress, cytokine release, and immune cell infiltration, appear integral to both NVAF onset and persistence [4,5].

Inflammatory markers, such as C-reactive protein (CRP), have been linked to more severe NVAF outcomes [6,7]. Recently, emerging composite indexes including the systemic inflammation response index (SIRI), neutrophil-to-lymphocyte ratio (NLR), platelet-to-lymphocyte ratio (PLR), and the systemic immune-inflammation index (SII) have attracted increasing attention for their potential to capture broader, dynamic inflammatory pathways [8,9,10]. These indexes may offer enhanced predictive insight by combining multiple hematologic parameters, reflecting not only short-lived immune responses but also the chronic inflammatory milieu that can fuel NVAF-related complications [6]. Moreover, preliminary evidence suggests these biomarkers might help identify patients at heightened risk of bleeding events and overall mortality, further underscoring their potential clinical significance [10,11].

Whether these markers refine or augment standard clinical tools, such as CHA_2_DS_2_-VASc and HAS-BLED, remains uncertain. Established scores predominantly focus on clinical risk factors like heart failure, age, and prior stroke, but may not fully account for evolving inflammatory processes that contribute to NVAF burden [12]. Recent modifications to conventional risk stratification models for NVAF have led to the introduction of the CHA_2_DS_2_-VA score, an updated parameter that excludes the female sex category from its calculation, in contrast to the traditional CHA_2_DS_2_-VASc score [13,14]. Recent studies suggest that, in the absence of other concomitant risk factors, female sex does not independently increase the risk of thromboembolic events, thereby challenging its inclusion as a weighted component in risk prediction models [15,16]. By omitting the sex variable, the CHA_2_DS_2_-VA score aims to refine risk stratification by more accurately identifying patients at elevated risk for stroke, potentially reducing the risk of overtreatment in women and enabling more individualized anticoagulation management strategies [13,17]. Integrating composite indexes like NLR, PLR, and SII into CHA_2_DS_2_-VA score could potentially optimize anticoagulation strategies by highlighting individuals more susceptible to adverse outcomes. Early intervention or more aggressive treatment regimens might then be selectively applied to these higher-risk patients, improving their long-term prognosis and reducing hospitalizations. Therefore, the aim of this observational case–control study was to evaluate the ability of NLR, PLR, and SII, as well as the CHA_2_DS_2_-VA score, to detect the occurrence of NVAF in patients.

## 2. Patients, Methods, and Ethics Committee Approval

### 2.1. Patient Selection and Methods

This observational case–control study was conducted on 330 patients with NVAF, and 201 individuals without NVAF as a control (NSR) group at Amasya University, Faculty of Medicine, Department of Cardiology outpatient clinic.

Patients who were under 18 years of age; being pregnant or diagnosed with atrial fibrillation due to heart valve disease such as moderate to severe mitral stenosis, mechanical mitral valves, bioprosthetic valves, or mitral valve repair; patients with acute or chronic infectious disease; patients being treated with immunosuppressive drugs or patients with known or suspected cancer; patients with liver failure, hepatitis, kidney failure, arthritis, systemic diseases; and patients with a history of injury or surgery within 2 months were excluded from study.

After obtaining consent from the patient diagnosed with NVAF, the patient’s laboratory tests at the time of diagnosis were noted; the patient was taken for additional evaluation at the cardiology clinic; the patient’s physical examination was performed; electrocardiography and transthoracic echocardiography were applied to each patient.

During the same period, participants also began recruiting for the control group for the study. Exclusion criteria for the control group were patients younger than 18 years of age; diagnosed with AF at any time point; patients with acute decompensated heart failure (HF); acute coronary syndromes; pulmonary embolism; acute renal failure; coagulation disorders; severe valvular hearth disease (moderate mitral stenosis and all other serious valve diseases and prosthetic valve disease); severe anemia; active thyroid disorders; pregnancy; malignancy; epilepsy; major surgery within the preceding two months; and active infection and/or sepsis. After obtaining consent from the participant, relevant laboratory tests were performed, and each participant’s physical examination was performed similarly to the NVAF patient group; electrocardiography and transthoracic echocardiography were applied to each participant.

A 12-lead ECG was taken with all participants in the supine position at rest.

Echocardiographic evaluation was performed using a PHILIPS EPIQ 7G (is manufactured by Philips Healthcare in Andover, Massachusetts, USA) ultrasound system color Doppler echocardiography device in the left decubitus position for all participants. Ejection fractions (EFs) of all participants were measured using the modified Simpson’s method as per the American Society of Echocardiography and the European Society of Cardiovascular Imaging criteria [18].

### 2.2. Definitions

AF is reflected on the surface electrocardiogram (ECG) by the absence of discernible and regular P waves, and irregular activation of the ventricles. This results in no specific pattern to RR intervals, in the absence of an atrioventricular block [19]. We included patients diagnosed with permanent AF in the study of patients aged over 18 years.

Diagnosis of non-valvular atrial fibrillation in the absence of moderate to severe mitral stenosis or mechanical heart valves, bioprosthetic valves, or mitral valve repair. The definition of AF by temporal pattern is permanent AF which no further attempts at restoration of sinus rhythm are planned, after a shared decision between the patient and physician [19].

We used the 2023 ESC Guidelines for the management of cardiovascular disease in patients with diabetes criteria, including fasting glucose, 2 h glucose (during the glucose tolerance test), random glucose, and glycated hemoglobin (HbA1c) to diagnose diabetes mellitus (DM) [20]. Hypertension is defined according to the 2024 ESC Guidelines for the management of elevated blood pressure and hypertension as a confirmed office systolic BP of ≥140 mmHg or diastolic BP of ≥90 mmHg. For this diagnosis to be made, confirmation is recommended with out-of-office measurements (HBPM or ABPM) or at least one repeat office measurement at a subsequent visit, or those who were previously diagnosed with hypertension and started treatment were considered hypertensive [21].

The presence of stroke/TIA in patients was determined based on the criteria outlined in “A consensus report from the European Society of Cardiology Cardiovascular Round Table” [22].

Vascular disease was defined as coronary artery disease, including prior myocardial infarction, angina, history of coronary revascularization (surgical or percutaneous), and evident CAD on angiography or cardiac imaging or peripheral vascular disease (PVD), including the following: intermittent claudication, prior revascularization for PVD, percutaneous or surgical intervention on the abdominal aorta, and complex aortic plaque (defined as mobility, ulceration, pedunculation, or thickness ≥ 4 mm) on imaging [23,24,25].

Demographic characteristics (age and gender), blood and serum parameters (e.g., total cholesterol, low-density lipoprotein (LDL), high-density lipoprotein (HDL), triglyceride (TG), C reactive protein (CRP), serum albumin, creatinine (Cr), alanine aminotransferase (ALT), aspartate aminotransferase (AST), hemoglobin (HG), uric acid, Na^+^, K^+^, white blood cell counts); LVEF values and presence of diabetes mellitus (DM), hypertension (HT), cerebrovascular accident (SVA), Vascular Disease (VD), were analyzed. CHA_2_DS_2_-VA score [C: congestive HF or left ventricular systolic dysfunction, H: hypertension, A: ≥75 years, D: diabetes mellitus, S: previous stroke, V: vascular disease, A: 65–74 years] scores of both groups were calculated.

Inflammatory indexes defined and calculated as SII, the systemic immune-inflammation index (platelet count x neutrophil count/lymphocytes count); SIRI, the systemic inflammation response index (neutrophil count × monocyte count/lymphocyte count; BAR, Blood Urea Nitrogen to Serum Albumin (g/L) Ratio; NLR, neutrophil count/lymphocytes count ratio; CAR, C-reactive protein to albumin (g/L) ratio; UAR, Uric acid to albumin (g/L) ratio; PNI, prognostic nutritional index (albumin level (g/L) + 0.005 × lymphocyte count); MHR, the monocyte count/high-density lipoprotein cholesterol (mg/dL) ratio; NHR, neutrophil count-to-high-density lipoprotein cholesterol (mg/dL) ratio; PLR, the platelet count/lymphocyte count ratio; MLR, monocyte count/lymphocyte count ratio; LCR, lymphocyte count to C-reactive protein ratio; LMR, lymphocyte count/monocyte count ratio; TyG, The triglyceride–glucose index (Ln [fasting triglycerides (mg/dL) × fasting glucose (mg/dL)/2]); TG/HDL, triglycerides (mg/dL) to high-density lipoprotein cholesterol (mg/dL) ratio.

### 2.3. Ethics Committee Approval

The study was approved by the ethics committee of the Amasya University Rectorate Non-Interventional Clinical Research Ethics Committee; Board Decision Number: E-30640013-050.04-246857.

### 2.4. Statistical Analysis

The statistical analysis of the study data was performed using the SPSS software (Version 22.0, SPSS, Chicago, IL, USA). Continuous variables were expressed as mean ± standard deviation (SD), while categorical variables were presented as frequencies and percentages. The Kolmogorov–Smirnov test was used to assess the distribution pattern of the variables. For group comparisons, both the *t*-test and chi-square test were employed. *p* < 0.05 was considered statistically significant.

For the intergroup statistical analysis, categorical variables were compared using the χ2 test or Fisher’s exact test, while continuous variables were analyzed using either the *t*-test or Mann–Whitney U test, based on the normality of distribution. To explore the independent association between inflammatory markers and NVAF, multivariate logistic regression analysis was employed. Odds ratios (ORs) and 95% confidence intervals (CIs) were calculated for each associated variable. Furthermore, a receiver operating characteristic (ROC) curve analysis was conducted to assess the sensitivity and specificity of the CHA_2_DS_2_-VA score for detecting NVAF. A *p*-value of 0.05 was considered statistically significant across all analyses. Optimal cut-off value for the CHA_2_DS_2_-VA score _score_ was determined using ROC curves, with the area under the curve (AUC) and 95% CI calculated for the marker. All statistical analyses were performed using R software version 3.6.3 (R Foundation for Statistical Computing, Vienna, Austria), MedCalc Programme (trial version), and IBM SPSS Statistics version 26.0. A two-tailed *p*-value of 0.05 was deemed significant in all analyses.

## 3. Results

The mean age of the study group was 77.70 ± 5.94 years. The mean age in the NVAF group (79.29 ± 6.64 years) was significantly higher compared to the NSR group (75.09 ± 3.14 years) (*p* < 0.001). Gender distribution among the groups was similar (*p* = 0.465; Table 1). There was no significant difference in gender distribution between the NVAF group (188 females, 142 males) and the NSR group (121 females, 80 males) (*p* = 0.465).

The prevalence of hypertension (HT) in the NVAF group (95.2%) was significantly higher than in the NSR group (81.1%) (*p* < 0.001). Similarly, the prevalence of cerebrovascular disease (CVD) in the NVAF group (17.9%) was significantly higher than in the NSR group (5.5%) (*p* < 0.001). However, no significant difference was observed between the groups regarding the prevalence of diabetes mellitus (DM) (*p* = 0.80).

The mean LVEF of the patient group was 48.81 ± 10.34, and the mean LVEF of the control group was 58,59 ± 5.31 (*p* < 0.0001; Table 1).

The incidence of vascular disease in the patient group was (%) 9.1 (30/330), and the incidence of vascular disease in the control group was (%) 3.0 (6/195) (*p*-value: 0.007; Table 1).

The mean CHA_2_DS_2_-VA score of the patient group was 3.66 ± 1.14, and the mean CHA_2_DS_2_-VA score of the control group was 2.10 ± 0.98 (*p* < 0.0001; Table 1).

The CHA_2_DS_2_-VA score was significantly higher in the NVAF group (3.66 ± 1.14) compared to the NSR group (2.10 ± 0.98) (*p* < 0.001). In the NVAF group, the proportions of patients with CHA_2_DS_2_-VA scores of 1 and 2 were 0.9% and 10.9%, respectively, whereas in the NSR group, these rates were 22.9% and 51.7%, respectively. The proportions of patients with CHA_2_DS_2_-VA scores of 3 and 4 in the NVAF group were 37.6% and 31.8%, respectively, while in the NSR group, they were 19.4% and 4.0%, respectively. In the NVAF group, 12.1% and 4.8% of patients had CHA_2_DS_2_-VA scores of 5 and 6, respectively, compared to 2.0% and 0.0% in the NSR group. Additionally, 1.2% and 0.6% of patients in the NVAF group had CHA_2_DS_2_-VA scores of 7 and 8, respectively, whereas no patients in the NSR group had scores of 7 or 8. These findings indicate that the stroke risk detected by the CHA_2_DS_2_-VA score was higher in the NVAF group.

Glucose, Cr, ALT, Na^+^, K^+^, TG, Hgb, WBC, PLT, and MPV levels were similar in the control and patient groups (Table 2). BUN, AST, CRP, uric acid, neutrophil, and monocyte levels were significantly higher in the patient group compared to the control group (*p* < 0.001; Table 2). While albumin, total cholesterol, HDL, LDL (*p*-value: 0.001), and lymphocyte levels were significantly higher in the control (NSR) group compared to the NVAF group (*p* < 0.001; Table 2). On the other hand, while TyG levels were similar in both groups, TG/HDL, SII, SIRI, BAR, UAR, MHR, NLR, PLR, and CAR levels were significantly higher in the patient group compared to the control group (*p* < 0.001; Table 2). Additionally, PNI, LCR, and LMR levels were significantly higher in the control (NSR) group compared to the NVAF group (*p* < 0.001; Table 2).

In the NVAF group, most inflammatory markers were found to be significantly higher compared to the NSR group. CRP levels were significantly elevated in the NVAF group compared to the NSR group (*p* < 0.001). Similarly, the NLR was significantly higher in the NVAF group than in the NSR group (*p* < 0.001). The PLR was also significantly elevated in the NVAF group (263.84 ± 130.7) compared to the NSR group (*p* < 0.001).

The SII was significantly higher in the NVAF group than in the NSR group (*p* < 0.001). Furthermore, LCR was significantly lower in the NVAF group (0.14 ± 0.06) compared to the NSR group (*p* < 0.001). Similarly, the prognostic PNI was significantly lower in the NVAF group than in the NSR group (*p* < 0.001), mirroring the trend observed with the LCR. These findings indicate that systemic inflammation is more pronounced in patients with NVAF.

### 3.1. Multivariate Analysis of CHA_2_DS_2_-VA Score and Hematological Indexes in NVAF Group

Table 3 shows multivariate analyses of risk markers for NVAF. A multivariate logistic regression analysis was conducted to evaluate the independent detective value of inflammatory markers and clinical parameters in the association between the NVAF and NSR groups. According to the results of the analysis, age (OR: 1.15, 95% CI: 1.14–1.20, *p* < 0.001), the presence of cerebrovascular disease (OR: 3.76, 95% CI: 1.92–7.34, *p* < 0.001), and vascular disease (OR: 3.25, 95% CI: 1.32–7.95, *p* = 0.010) were identified as significant risk factors for the development of NVAF. The CHA_2_DS_2_-VA score demonstrated the strongest association with NVAF (OR: 5.55, 95% CI: 4.11–7.50, *p* < 0.001), further emphasizing the importance of this score in risk stratification.

Among the inflammatory markers, the SII (OR: 1.01, 95% CI: 1.01–1.02, *p* < 0.001), SIRI (OR: 1.87, 95% CI: 1.65–2.12, *p* < 0.001), PLR (OR: 1.04, 95% CI: 1.03–1.04, *p* < 0.001), and NLR (OR: 4.02, 95% CI: 2.05–8.03, *p* < 0.001) were all found to be significantly associated with the presence of NVAF. These findings underscore the important role of both clinical risk factors and systemic inflammation in the pathogenesis of NVAF.

Table 3 demonstrates that, in multivariate logistic regression analysis, inflammatory markers possess independent risk markers for the development of NVAF and suggest that integrating these markers into existing risk scoring systems may enhance clinical decision-making processes. In particular, composite indexes such as SII, SIRI, PLR, and NLR may serve as potential biomarkers for the early diagnosis and risk stratification of NVAF. Finally, these results indicate that the CHA_2_DS_2_-VA score can be used with high accuracy to identify patients with NVAF (Table 3).

### 3.2. ROC Curve Analyses of CHA_2_DS_2_-VA Score in NVAF

**Figure 1:**
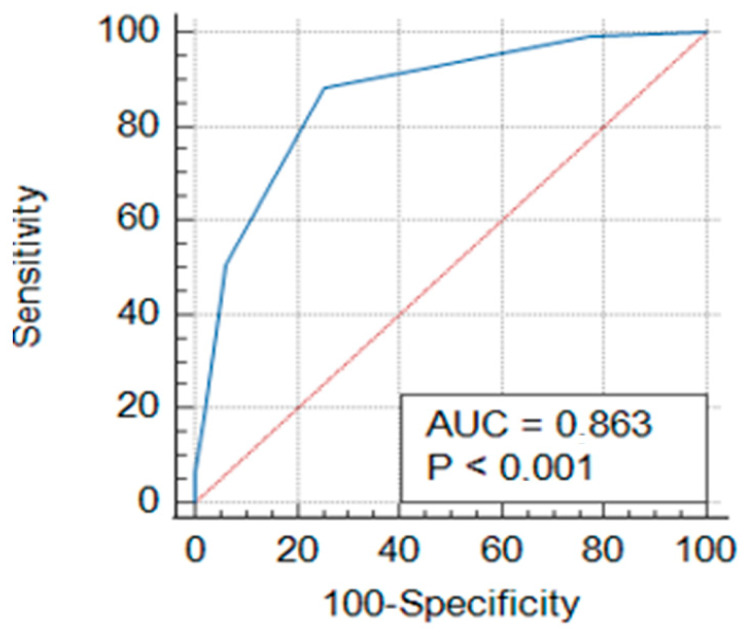
Receiver operating characteristic (ROC) curve of CHA_2_DS_2_-VA score.

Figure 1 and Table 4 show the ROC curve and prognostic accuracy of the CHA_2_DS_2_-VA score.

The performance of the CHA_2_DS_2_-VA score in detecting NVAF diagnosis was evaluated using receiver operating characteristic (ROC) curve analysis. Figure 1 shows that the area under the curve (AUC) value for the CHA_2_DS_2_-VA score was found to be 0.863 (*p* < 0.001). The cut-off value for the CHA_2_DS_2_-VA score was determined as 2. At this threshold, the sensitivity of the score was calculated to be 88.18%, and the specificity was 74.63% (Table 4).

## 4. Discussion

The pathophysiology underlying NVAF is multifaceted, encompassing structural, electrical, and inflammatory mechanisms. Among these, systemic inflammation has been increasingly recognized as a critical contributor to both the initiation and perpetuation of atrial fibrillation (AF) [26]. Elevated levels of inflammatory biomarkers, such as CRP, interleukin-6 (IL-6), and tumor necrosis factor-alpha (TNF-α), have been consistently associated with the occurrence and prognosis of AF, underscoring the role of inflammatory processes in atrial remodeling, fibrosis, and thrombogenesis [27,28].

Recent research has shifted toward exploring readily available and cost-effective hematological parameters as potential indicators of systemic inflammation and predictors of NVAF risk [10,11]. Specifically, NLR, PLR, SII, and SIRI have emerged as promising biomarkers due to their ease of measurement and integration into routine clinical practice. These indexes not only reflect inflammatory status but also provide insights into immune dysregulation and prothrombotic states, which are integral to NVAF pathogenesis [10].

Our results demonstrated that NLR, SII, SIRI, PLR, and CHA_2_DS_2_-VA scores were significantly higher in the NVAF group than in the NSR group. In our multivariate analysis, risk factors were identified in the NVAF group. In particular, the CHA_2_DS_2_-VA score showed the strongest association with NVAF (OR: 5.55, 95% CI: 4.11–7.50), with each incremental increase in the score markedly elevating the risk. Additionally, well-known risk factors, including age, hypertension, and previous cerebrovascular events, were significantly associated with NVAF. Furthermore, an elevated NLR increased the odds of NVAF almost fourfold (OR: 4.02, 95% CI: 2.05–8.03). Similarly, SIRI was also identified as a risk factor for NVAF (OR: 1.87, 95% CI: 1.65–2.12), while PLR (OR: 1.04, 95% CI: 1.03–1.04) and SII (OR: 1.01, 95% CI: 1.01–1.02) were statistically significantly detected with more modest risk increases. Considering the broad range of these parameters, markedly elevated PLR or SII may indicate a substantially increased NVAF risk. Collectively, these findings suggest that both traditional clinical risk factors and systemic inflammation contribute complementarily to the pathogenesis of NVAF.

The association between AF and systemic inflammation has long been reported in the literature [29]. Previous studies have shown that inflammatory markers such as CRP and interleukin-6 are closely related to both the presence and duration of AF, with inflammatory cell infiltrates being detected in atrial tissue specimens [29]. Inflammation is considered to trigger atrial remodeling through pathways involving oxidative stress, apoptosis, and fibrosis, thereby predisposing to the development of AF [30,31]. Moreover, inflammation may also contribute to the thrombotic complications associated with AF by promoting endothelial dysfunction, platelet activation, and coagulation [32].

Regarding the neutrophil-to-lymphocyte ratio, a recent meta-analysis reported that elevated NLR is associated with increased AF recurrence and stroke risk in patients with AF [33]. Additionally, higher NLR values have been linked to left atrial thrombus formation, emphasizing its value as a prognostic biomarker [33]. Our finding of significantly elevated NLR in the NVAF group further supports the clinical importance of NLR in risk stratification. Similarly, the platelet-to-lymphocyte ratio (PLR) is recognized as an indicator of both inflammatory and thrombotic states, which are mechanistically involved in AF pathogenesis. The high PLR values in the NVAF group of our study align with reports in the literature indicating PLR as an independent predictor of AF recurrence post-ablation and incorporating PLR into conventional risk scores enhances predictive accuracy for AF recurrence, as reflected by improved model discrimination [34].

The systemic immune-inflammation index (SII) emerged as another significant parameter in our work. SII, which integrates neutrophil, platelet, and lymphocyte counts, has been validated as a prognostic marker for cardiovascular events. Recent studies have highlighted that elevated SII levels in NVAF patients are linked to a higher risk of left atrial thrombus formation [19]. For instance, one study found that an SII value above 693 predicted left atrial thrombus with 71.6% sensitivity and 71.7% specificity, suggesting that SII may be as effective as—or even superior to—NLR and PLR in certain clinical settings [20]. Accordingly, the high SII levels observed in our NVAF cohort further reinforce its role as a comprehensive marker that reflects both inflammatory and prothrombotic states [21].

Regarding SIRI, although available data are more limited, our findings are in line with emerging evidence. SIRI reflects the balance between pro-inflammatory (neutrophils and monocytes) and regulatory (lymphocytes) immune components, and its significant elevation in NVAF patients in our study is consistent with observations in ischemic stroke populations where patients with concomitant AF demonstrated much higher SIRI levels (with an OR for log-SIRI approximating 6.2) [22]. Such findings underscore the potential of SIRI to serve as an indicator of the intense inflammatory processes that may underlie AF pathogenesis [23].

Traditionally, the CHA_2_DS_2_-VASc has been used to predict stroke, major bleeding, and mortality in NVAF patients; however, it relies solely on clinical demographic and comorbidity data without reflecting the inflammatory processes [35,36]. Our study found that while a high CHA_2_DS_2_-VA is a strong independent risk marker of NVAF (with each additional point conferring significant risk), the inflammatory indexes, particularly NLR and SII, add prognostic value beyond the clinical score. This observation is consistent with the recent literature that underlines the limitations of traditional risk scores and emphasizes the need to incorporate inflammatory biomarkers into risk stratification models [37,38,39]. Several studies suggested that including biomarkers in conventional risk scores can improve the model’s discriminative power [40]. Therefore, in predicting key clinical outcomes such as stroke, major bleeding, and mortality, integrating inflammatory indexes like NLR, PLR, and SII alongside the CHA_2_DS_2_-VA may offer a more precise risk stratification [41]. In addition, our results showed that factors such as age, history of cerebrovascular events, vascular disease, and CHA_2_DS_2_-VA, in addition to NLR, PLR, SII, and SIRI, were significantly associated with increased NVAF risk. These results underscore that the inflammatory response plays a critical role in the pathogenesis of NVAF beyond the traditional clinical risk factors.

Although our study has several strengths, including its cross sectional design, adequate sample size, and comprehensive laboratory analyses, it also has several limitations. First, despite the prospective approach, our analysis represents a cross sectional comparison between NVAF patients and a control group; therefore, it is not fully clear whether the elevated inflammatory indexes are a cause or a consequence of AF. Since AF itself or its associated comorbidities may affect inflammation levels, caution is warranted in inferring a causal relationship [42]. Long-term prospective cohort studies that measure inflammatory indexes prior to the onset of AF are needed to clarify this issue. Second, as the study was conducted at a single center, the generalizability of the results to different geographic regions or ethnic groups may be limited. Third, the inflammatory markers can be influenced by various factors such as chronic inflammatory conditions, acute infections, or medications, such as corticosteroids, which may affect the specificity of the measurements. Fourth, the intercorrelation among indexes like NLR, PLR, SII, and SIRI could introduce challenges in determining the most prognostically valuable marker when used together; thus, future studies may consider developing a composite score that integrates these measures. Additionally, left atrial strain parameters, particularly left atrial strain rate (LASr)—which have demonstrated incremental prognostic value in NVAF patients—were not evaluated in this study [43,44]. Recent data indicate that LASr is strongly associated with both myocardial fibrosis and inflammation and may further improve risk stratification in NVAF populations [45]. Nonetheless, the detailed evaluation of inflammatory indexes and their independent detection values for NVAF provided by our study represents a significant contribution to the literature.

## 5. Conclusions and Recommendations

This study is one of the first observational case–control study investigations to systematically evaluate the role of inflammatory processes in the pathogenesis of NVAF and to demonstrate the prognostic value of next-generation inflammatory indexes (NLR, PLR, SII, SIRI) when integrated with the CHA_2_DS_2_-VA score. Our findings fill a gap in the existing literature by providing strong evidence that these indexes can significantly enhance risk stratification in NVAF patients compared to traditional scoring systems. In particular, composite indexes such as SIRI and SII highlight the potential for an inflammation-focused paradigm in personalized treatment strategies.

The study offers a comprehensive cross sectional analysis of the diagnostic and prognostic utility of inflammatory indexes (NLR, PLR, SII, SIRI) alongside the CHA_2_DS_2_-VA score in NVAF patients. We demonstrated that these indexes are significantly associated with both the presence and severity of NVAF. Notably, NLR (OR = 4.02) and the CHA_2_DS_2_-VA score (OR = 5.55) exhibited the strongest associations, underscoring the critical role of inflammation in NVAF pathogenesis.

We recommend incorporating inflammatory indexes such as NLR, PLR, and SII together with the CHA_2_DS_2_-VA score into the routine evaluation of NVAF patients. This combined approach may be particularly valuable for the early identification of high-risk individuals and for optimizing anticoagulation strategies. Future studies should focus on developing composite risk scores that integrate inflammatory biomarkers (for example, a CHA_2_DS_2_-VA-Inflammation Score) and should validate these models in independent cohorts to assess their potential for improving clinical outcomes. Additionally, longitudinal studies and randomized controlled trials targeting inflammation (e.g., with IL-6 inhibitors) are needed to elucidate the causal role of inflammation in NVAF. Also, replication of these findings in multicenter studies will be essential to increase the generalizability of the results.

## Figures and Tables

**Table 1:** Participants’ demographic and disease data.

Variable	NVAF (*n* = 330)	NSR (*n* = 201)	*p*-Value
Sex (F/M)	188/142	121/80	0.465
LVEF (%)	48.81 ± 10.34	58.59 ± 5.31	*p* < 0.001
HT (+/−)	314/16	163/38	*p* < 0.001
Age (years)	79.29 ± 6.64	75.09 ± 3.14	*p* < 0.001
DM (+/−)	72/258	33/168	0.800
CVA (+/−)	59/271	11/190	*p* < 0.001
VD (+/−)	30/330	6/195	0.007
CHA_2_DS_2_-VA score	3.66 ± 1.14	2.10 ± 0.98	*p* < 0.001

F: Female; M: Male; LVEF: Left ventricular ejection fraction (%); HT: Hypertension; DM: Diabetes mellitus, CVA: Cerebrovascular accident; VD: Vascular Disease; CHA_2_DS_2_-VA score [C: congestive HF or left ventricular systolic dysfunction, H: hypertension, A: ≥75 years, D: diabetes mellitus, S: previous stroke, V: vascular disease, A: 65–74 years].

**Table 2:** Participants’ laboratory and inflammatory index data.

Variable	NVAF (*n* = 330)	NSR (*n* = 201)	*p*-Value
Glucose (mg/dL)	109.73 ± 35.70	106.55 ± 38.53	0.335
BUN (mg/dL)	44.35 ± 8.86	34.26 ± 7.23	*p* < 0.001 *
Cr (mg/dL)	0.92 ± 0.22	0.88 ± 0.20	0.015
Albumin (g/L)	32.66 ± 5.59	41.53 ± 2.63	*p* < 0.001
AST (U/L)	23.64 ± 8.37	20.62 ± 5.96	*p* < 0.001 *
ALT (U/L)	15.96 ± 7.97	16.51 ± 6.26	0.402
Na^+^ (mmol/L)	139.04 ± 3.31	139.39 ± 2.98	0.184
K^+^ (mmol/L)	4.33 ± 0.48	4.38 ± 0.40	0.192
CRP (mg/L)	7.79 ± 1.65	2.42 ± 1.13	*p* < 0.001 *
TG (mg/dL)	116.88 ± 50.72	123.72 ± 43.46	0.113
TC (mg/dL)	153.92 ± 38.02	176.37 ± 37.64	*p* < 0.001
HDL (mg/dL)	35.22 ± 7.88	49.86 ± 12.43	*p* < 0.001 *
LDL (mg/dL)	99.14 ± 28.93	108.33 ± 30.07	0.001 *
Uric acid (mg/dL)	7.77 ± 1.83	5.03 ± 0.98	*p* < 0.001
WBC (10^3^/μL)	7.14 ± 1.3	7.15 ± 1.26	0.933
HGB (g/dL)	12.96 ± 1.53	13.11 ± 1.55	0.266
PLT (10^3^/μL)	243.12 ± 72.44	234.94 ± 56.38	0.172
MPV	10.86 ± 17.22	10.37 ± 0.75	0.683
Neutrophil (10^3^/μL)	7.51 ± 1.51	4.47 ± 1.24	*p* < 0.001 *
Lymphocyte (10^3^/μL)	1.05 ± 0.40	2.1 ± 0.53	*p* < 0.001 *
Monocyte (10^3^/μL)	0.76 ± 0.37	0.57 ± 0.15	*p* < 0.001 *
TyG	8.63 ± 0.55	8.68 ± 0.46	0.265
TG/HDL	3.50 ± 1.75	2.63 ± 1.12	*p* < 0.001 *
SII	1952 ± 1035	521.17 ± 206.37	*p* < 0.001 *
SIRI	6.15 ± 4.43	1.28 ± 0.58	*p* < 0.001 *
BAR	1.40 ± 0.38	0.82 ± 0.18	*p* < 0.001 *
UAR	0.24 ± 0.07	0.12 ± 0.02	*p* < 0.001 *
PNI	32.66 ± 5.59	41.54 ± 2.63	*p* < 0.001 *
MHR	0.022 ± 0.014	0.012 ± 0.004	*p* < 0.001 *
NHR	0.22 ± 0.07	0.09 ± 0.03	*p* < 0.001 *
NLR	8.29 ± 4.5	2.22 ± 0.76	*p* < 0.001 *
PLR	263.84 ± 130.7	118.45 ± 39.11	*p* < 0.001 *
MLR	0.81 ± 0.56	0.28 ± 0.09	*p* < 0.001 *
LCR	0.14 ± 0.06	1.20 ± 1.14	*p* < 0.001 *
CAR	0.24 ± 0.07	0.05 ± 0.028	*p* < 0.001 *
LMR	1.56 ± 0.68	3.89 ± 1.28	*p* < 0.001 *

BUN: Blood Urea Nitrogen; Cr: Creatinine; AST: Aspartate Aminotransferase; ALT: Alanine Aminotransferase; Na^+^: Sodium; K^+^: Potassium; CRP: C-Reactive Protein; TG: Triglycerides; TC: Total Cholesterol; HDL: High-Density Lipoprotein; LDL: Low-Density Lipoprotein; WBC: White Blood Cell count; HGB: Hemoglobin; PLT: Platelet Count; MPV: Mean Platelet Volume; TyG: The triglyceride-glucose; TG/HDL: triglyceride to high-density lipoprotein cholesterol; SII: the systemic immune-inflammation ındex; SIRI: the systemic inflammation response index; BAR: Blood Urea Nitrogen to Serum Albumin Ratio; NLR: neutrophil/lymphocyte ratio; CAR: C-reactive protein to albumin ratio; UAR: Uric acid to albumin ratio; PNI: Prognostic Nutritional Index; MHR: the monocyte/high-density lipoprotein ratio; NHR: neutrophil-to-high-density lipoprotein cholesterol ratio; PLR: the platelet/lymphocyte ratio; MLR: monocyte/lymphocyte ratio; LCR: lymphocyte to C reactive protein ratio; LMR: lymphocyte/monocyte ratio. *: Values showing significant intergroup differences that do not conform to a normal distribution are marked with an asterisk; these comparisons were performed using the Mann–Whitney U test. The remaining values were compared using Student’s *t*-test.

**Table 3:** Multivariate logistic regression model for the association between NVAF group and NSR group.

Variable	Odds Ratio	(95% CI)	*p*-Value
Age (years)	1.15	1.14–1.20	*p* < 0.001
CVA	3.76	1.92–7.34	*p* < 0.001
VD	3.25	1.32–7.95	0.010
CHA_2_DS_2_-VA	5.55	4.11–7.50	*p* < 0.001
SII	1.01	1.01–1.02	*p* < 0.001
SIRI	1.87	1.65–2.12	*p* < 0.001
PLR	1.04	1.03–1.04	*p* < 0.001
NLR	4.02	2.05–8.03	*p* < 0.001

CVA: Cerebrovascular accident; VD: Vascular Disease; CHA_2_DS_2_-VA score [C: congestive HF or left ventricular systolic dysfunction, H: hypertension, A: ≥75 years, D: diabetes mellitus, S: previous stroke, V: vascular disease, A: 65–74 years]; SII: the systemic immune inflammation ındex; SIRI: the systemic inflammation response index; PLR: the platelet/lymphocyte ratio; NLR: neutrophil/lymphocyte ratio.

**Table 4:** ROC curve and prognostic accuracy of the CHA_2_DS_2_-VA score.

	AUC	Sensitivity (%)	Specificity (%)	Cut-Off	*p*-Value
CHA_2_DS_2_-VA score	0.863	88.18	74.63	2	*p* < 0.001

## Data Availability

Data are provided within the manuscript. If needed, the datasets generated and/or analyzed during the current study are available from the corresponding author upon reasonable request.
